# Parents’ and medical staff’s experience of adolescents with suicide-related behaviors admitted to a general hospital in China: qualitative study

**DOI:** 10.1186/s12888-021-03057-w

**Published:** 2021-01-28

**Authors:** Xi Fu, Jiaxin Yang, Xiaoli Liao, Yidong Shen, Jianjun Ou, Yamin Li, Runsen Chen

**Affiliations:** 1 National Clinical Research Center for Mental Disorders, Department of Psychiatry, and China National Technology Institute on Mental Disorders, The Second Xiangya Hospital, Central South University, Changsha, 410011 Hunan China; 2 Clinical Nursing Teaching and Research Section, The Second Xiangya Hospital, Central South University, Changsha, 410011 Hunan China; 3 XiangYa Nursing School, Central South University, Changsha, 410011 Hunan China

**Keywords:** Suicide-related behaviors, Adolescent, Medical staff, Parents

## Abstract

**Background:**

Currently, there is increasing awareness of suicide-related behaviors. Mental health services are a key location for assisting people with suicide-related behaviors. However, few studies focused on the evaluation and experience of the mental health care system from families and the medical staff’s perspective in China. The study aims to explore parents’ and the front-line medical staff’s experience of an adolescent with suicide-related behaviors admitted to the psychiatry department of a general hospital in China.

**Design:**

Qualitative study was employed in the study. Participants were recruited from a general hospital in China characterized by high levels in the Chinese mental health system.

**Methods:**

Semi-structured in-depth interviews were conducted exploring their experience and perceptions when an adolescent was admitted to the hospital. The theme analysis method is used for data analysis.

**Results:**

Participants expressed dissatisfaction in the psychiatric department. Other barriers in their work were identified, such as the shortage of staff and difficulties in caring or communicating with patients. Besides, the imperfect treatment system also contributes to the low satisfaction of patients and their families. Two themes and six subthemes were identified: 1) staff perceive patients with SRBs as difficult to engage (feelings of helplessness, the need for compassion, challenges of professional self-efficacy, the recommendations to the health care service); 2) parents not satisfied with the existing hospital services (doubt the hospitalization treatment and the advice to the health care service).

**Conclusion:**

This study found that insufficient staffing and lacking of systematic professional treatment models are the major challenges. We suggest increasing the input of mental health resources to expand and train the mental health service team and establish a complete set of a treatment model for SRBs.

## Background

Suicide-related behaviors (SRBs) [1] consisting of suicidal ideation, suicidal planning, and attempted suicide have become a serious cause of death worldwide for all age groups in the past 30 years. Data from the World Health Organization estimates that in 2017, the global number of suicide deaths was about 804,000, with an average of 11.4 suicides per 100,000 people [2]. Currently, suicide has become the third leading cause of death among youth aged 10–19 across the world [3], which makes suicide among young people a focus of global public health [4]. Self-harm is one of the strongest predictors of future suicide [5]. It is defined as the deliberate destruction of one’s body tissue not for suicide. Its common forms include cutting, scratching, and punching [6]. The data show that self-harm among adolescents is common, and they have a higher incidence rate of this behavior than any other age group [7]. In the United States, 17.58% of teenagers engaged in self-harm [8]; and in China, that statistic was even higher. Among middle school and high school students, the incidence of non-suicidal self-injury (NSSI) was 20.6% for males and 21.9% for females [9].

Extensive literature indicates that there are many predisposing factors for SRBs [10], including individual factors (heritable, demographic, and biological) [11, 12] and environmental factors (e.g., childhood abuse and adverse life events) [13]. Besides, SRBs are common in patients with mental illness [14–16]. Prior work has demonstrated that the most common risk factor for SRBs is mental illness among adolescents [17]. And not only that, SRBs in adolescents could affect their family and/or friends, which was known as “social contagion” [18]. A systematic review indicates that, the risk of suicide [19] and suicidal thoughts [20] was increased during the process after the survivors lost a significant other.

Currently, it is widely agreed that generic mental health services have important roles in contemporary service provision [21] and provides an opportunity for active intervention especially for serious SRBs. Unfortunately, previous studies demonstrated the low consultation rate of adolescents with SRBs [22]. The data shows that only 24% of individuals were diagnosed with a mental illness in the 4 weeks before they died [23]. Medical staff and parents played a vital role in the process of seeking help and getting treatment for adolescents with SRBs. Thus, it is crucial to understand their perceptions and experiences during the patients admitted to the hospital for treatment.

Previous research found that working with in-patients who are suicidal is a high demanding job with low autonomy and inadequate support [24]. Meanwhile, nurses who experienced inpatient suicide became stressed and showed excessive vigilance, which was not conducive to the patient or the practice [25]. However, most of the researches were conducted in developed countries. Few studies commented on the health care system in China. Therefore, we conducted one-to-one, in-depth interviews with the medical staff and parents of adolescents with SRBs to investigate their perceptions of the existing mental health care system. Qualitative research methods could help to gain a deeper understanding of an individual’s perceptions and thoughts, which is beneficial to uncover potential information [26, 27]. This study aims to do the following: 1) identify the existing problems related to SRBs in psychiatry departments, 2) provide directions for improving the Chinese mental health care system in this area, and 3) ultimately improve the relationship between parents and medical staff to better serve patients.

## Methods

### Sampling

Purposive and snowball sampling strategies were utilized to enable the maximum variation of identified participants through a gradual selection process. All participants were enrolled from a large tertiary hospital and included front-line medical staff (psychiatrists and psychiatric nurses) and the parents of adolescents exhibiting SRBs. Recruitment posters that described the study were placed in each psychiatric ward (a total of 6 wards) for a week. The inclusion criteria for medical staff incorporated the following: 1) working in a mental health setting for more than 1 month; 2) working with adolescents who are engaged in SRBs, and 3) the ability to complete the interview with normal intelligence and listening skills. The inclusion criteria for parents required a biological child who was engaged in SRBs and the ability to complete the interview with normal intelligence and listening skills. Participants with major physical illnesses (e.g. severe heart disease, liver and kidney disease) and parents who did not know that their children had SRBs were excluded. Participants had a week to contact us through the phone number listed on a poster. Meanwhile, written informed consent forms were issued, and participants were able to choose a time for the interview. Recruitment continued until the data quota had been reached. The study, conducted from April 2019 to August 2019, has gained the ethical approval of the Medical Ethics Committee of the Second Xiangya Hospital of Central South University (Ethics reference number: 2018048). Informed consent forms have been signed by all participants.

### Data collection

The study consisted of a 30–40-min, in-depth, semi-structured interview in an interview room in the psychiatric ward, which is a private space. The outline of the interview was based on relevant literature as well as consultation with psychiatrists and psychologists experienced in working with adolescents with SRBs. To enhance the credibility of the findings, before the formal interview, we conducted a pre-experiment to ensure the feasibility and completeness of the interview outline and make appropriate modifications. All of the interviews were conducted by XF and JXY and were audio-recorded. The following are some of the entries in the final, confirmed version of the interview outline.

For medical staff:

1. Are there any changes since you started working in psychiatry? If so, how do you think these changes affected your career?
2. Are there any challenges in your career?
3. How does working with patients who exhibit SRBs affect your life and work?
4. Do you have training opportunities related to the management of patients with SRBs? How often (the content of training)?
5. What skills (traits, skills, psychology, etc.) do you think are needed to do this job well?

For parents:

1. Did you persuade your child to go to the hospital?
2. What do you think of your child’s hospitalization experience?
3. What do you think of the medical staff? Are you satisfied with the treatment, doctors, and nurses? What area(s) needs to be improved?
4. Did you receive adequate medical services?

### Data analysis

Each recording was transferred into text within 48 h, and the text was checked with the participants. All identifiable text has been deleted, and the audio was destroyed after the transcription was completed. We used thematic analysis according to the six-step process of Braun and Clarke [28] to process the data, which was conducted by two researchers. Both researchers are in the field of psychiatry, and one of them has experience in qualitative research. The coding process was carried out in QSR NVivo version 11. The sub-themes were searching from codes that were identified by two authors. To ensure validation and reduce the bias of researchers, several discussions were conducted until all authors reached an agreement on the sub-themes.

## Results

In total, 34 participants completed the interview, consisting of 9 psychiatrists (3 females and 6 males), 10 psychiatric nurses (9 females and 1 male), and 15 parents (11 mothers and 4 fathers).

The professional experience of the psychiatrists ranges from 11 months to 8 years. Psychiatric nurses included qualified nurses (*n* = 9) and an advanced practice nurse (*n* = 1). The professional experience of nurses in the mental health setting ranges from 1 month to 15 years. Most of the psychiatrists (55.6%) and psychiatric nurses (60%) hold bachelor’s degrees (See Table 1).

**Table 1:** Demographic characteristics of psychiatrists and psychiatric nurses

Medical staff’s characteristics	*N* = 19
**Gender**	
Female	12(63.2%)
Male	7(36.8%)
**Education of psychiatrists**	
Bachelor’s degree	5(55.6%)
Master candidate	4(44.4%)
**Education of nurses**	
Vocational school	2(20%)
Bachelor’s degree	7(70%)
Master	1(10%)
**Professional experience of psychiatrists**	
0–5 years	7(77.8%)
5–10 years	1(11.1%)
Above 10 years	1(11.1%)
**Professional experience of nurses**	
0–5 years	6(60%)
5–10 years	1(10%)
Above 10 years	3(30%)

About 50% of parents have a high school education or above, and 73.3% of families live in cities and towns. Four participants were divorced, and 1 participant was widowed. Regarding the children, it was found that 12 adolescents had been diagnosed with non-psychotic major depression and 3 adolescents with non-psychotic bipolar disorder according to the International Classification of Diseases 10th Revision (ICD-10). They came to the hospital for treatment due to suicide attempts/behaviors or repeated self-harm. Among them, 10 adolescents had self-harm behaviors and 5 adolescents had suicidal behaviors or suicidal attempts (See Table 2). Two themes and six subthemes showed in Table 3.

**Table 2:** Demographic characteristics of parents

Parents characteristics	*N* = 15
**Age, years of child**	14.2(2.0;12–18)
**Residence**	
City	11(73.3%)
Countryside	4(26.7%)
**Education of father**	
Below high school	6(40.0%)
High school	3(20.0%)
Above high school	6(40.0%)
**Education of mother**	
Below high school	8(53.3%)
High school	3(20%)
Above high school	4(26.7%)
**Marital status of participants**	
Married	10(66.7%)
Divorce	4(26.7%)
Widowed	1(6.6%)

**Table 3:** Themes and subthemes

Themes	Sub-themes
**Staff perceive patients with SRBs as difficult to engage**	Feelings of helplessness
	The need for compassion
	Challenges of professional self-efficacy
	The recommendations to the health care service
**Parents not satisfied with the existing hospital services**	Doubt the hospitalization treatment
	The advice to the health care service

### Theme one: staff perceive patients with SRBs as difficult to engage

#### Feelings of helplessness

Many participants reported that they feel powerless, mainly because everyone’s disease is heterogeneous and the treatment is not effective for everyone. At times they feel that some of the treatments they do are futile. In addition, patients are not cooperative enough.

*“It's when you want to help a patient, but it feels like sometimes you can't help her, there is a feeling of powerlessness … ” (Nurse, p11)*

*“ … But sometimes they may have many strange reasons to hurt themselves. I don't know how to help him … ” (Nurse, p10)*

Other participants mentioned that their sense of achievement feels exceptionally low. Unlike patients with physical illness, some patients do not feel sick and therefore think they do not need treatment. Moreover, some treatments do not have an immediate effect on them, and their hospitalization is long but ineffective.

*"The patient doesn't understand and doesn't think he is sick, so his desire for treatment is not high, and then the doctor's sense of accomplishment is relatively low." (Psychiatrist, p5)*

*“ … I sometimes go to speak with patients, but there is no obvious effect for many times, and the patient's condition is not good. Then he couldn't understand what you were saying and couldn't follow your instructions, so I felt frustrated … ” (Nurse, p10)*

A few participants felt distrustful, making it difficult for them to communicate effectively with patients and gain a deeper understanding of what parents really think.

*“ … They don't want to communicate with people he doesn't trust, so they don't tell us exactly what he thinks. We can't collect all the information, and what we know is one-sided … ” (Psychiatrist, p1)*

*“ … Mistrust, especially at the beginning of hospitalization, because the effect of the drug has not yet been achieved, they think that the treatment may not be effective, so they will not trust us at that time … ” (Psychiatrist, p2)*

#### The need for compassion

Most participants mentioned that medical staff with patience, love, a calm personality, and a certain degree of discrimination and empathy are more qualified for working with SRBs adolescents.

*“ … I think this job requires a well-balanced personality, otherwise the problem will not be solved, and it will be more serious … ” (Nurse, p13)*

*“ … should be stronger inside and have the ability to distinguish, otherwise you will be led by the patient. Love and empathy are also required.” (Nurse, p11)*

#### Challenges of professional self-efficacy

Many participants said that working with teenagers who display SRBs requires strong communication and professional skills, and mastering these skills is a challenge.

*“ … We have to learn communication skills, and then learn some psychological counseling methods to empathize with patients, which is very difficult to learn … ” (Nurse, p16)*

*“ … I don't think I have enough ability. I have too much professional knowledge to learn … ” (p10)*

In addition, due to the recurrence of diseases, getting along with patients will encounter some challenges. There are many uncertain factors and the risk of accidents is high.

*“ … Compared with other patients, these patients have a higher risk of suiciding … ” (Psychiatrist, p4)*

*“ … For some patients, they don't want to talk to us at all, and they even don’t want to nod and shake head. I just feel like I can't get into his heart … ” (Nurse, p11)*

*“ … The patient may feel that I do not understand him or that the age gap is large … ” (Nurse, p12)*

A few participants mentioned that the lack of social recognition and acceptance of psychiatric patients is a major challenge. It is difficult for these patients to return to society after discharged.

*“ … The whole society still has a certain prejudice against mental illness. They cannot get social approval and support … ” (Psychiatrist, p3, p4)*

#### The recommendations to the health care service

Many participants mentioned that the department is understaffed, so there is less time to communicate with patients and their families, and the attention to patients may not be timely enough. They suggested increasing staffing and optimizing the division of labor.

*“ … There are too many patients in the department and too few medical staff. When a patient appear s suicide or injures himself, we sometimes fail to detect it in time … .” (Psychiatrist, p6)*

*“ … We do not have much time to communicate with patients and solve some of their psychological problems. Understaffing is a factor, and the second may be the division of labor is not optimized … ” (Nurse, p14)*

In terms of treatment, it is recommended that a complete set of treatment modes specifically for SRBs be developed with individualized treatment methods. In addition, at present, psychiatrists and nurses are mainly responsible for treatment tasks, and psychotherapists are in great need.

*“ … For self-harm and suicide, there is currently no perfect system of treatment mode. The main treatment now is drug treatment and communication, etc. It still has a lot of room for development … ” (Psychiatrist, p1)*

*“ … I think there is a need for personalized treatment plans because the group therapy we are doing now is similar to health education … ” (Nurse, p10)*

*“... Our hospital also has psychotherapy, but it is usually implemented by nurses rather than professional psychotherapists. Some nurses also do well, but there is still a mixture of the good and the bad in the area of psychotherapy … ” (Psychiatrist, p1)*

Some participants believed that supervision and training on SRBs are also needed. Communication skills and psychological intervention methods could be taught more professionally, which could help to dispel their negative emotions.

*“ … I think we need a professional psychotherapist to supervise us. Now we are just exploring some communication skills and psychological intervention methods ourselves … ” (Nurse, p12)*

*“ … I think more training on self-harm can be carried out in the future … ” (Nurse, p13)*

### Theme two: parents not satisfied with the existing hospital services

#### Doubt the hospitalization treatment

Most participants reported that medical staff were busy and communicated little with them and their children; therefore, their understanding of childhood mental health diseases and the treatment mechanisms were limited. Furthermore, it was felt that the medical staff focused on patients with more severe illnesses, and patients with mild illnesses were not treated equally.

*“ … I feel like they just come in the morning and ask if they have any questions. No one went to chat with my child or to communicate with her … ” (Mother, p23)*

*“ … Psychiatrists and nurses don't make enough rounds and careless. I think they can communicate more with patients and enlighten their minds … ” (Mother, p24)*

Some participants doubt the intervention due to the severe side effects of the drug. Furthermore, a few participants believed that their children’s behavior is not a natural manifestation but a response after treatment.

*“...After taking this medicine, she has obvious symptoms of nausea and vomiting...” (Mother, p38)*

*“...I feel like she's a little more excited now. Because she wouldn't do that kind of intimate behavior to me before, but she would do it deliberately now...” (Mother, p34)*

Other participants mentioned that there are limited treatments administered in hospitals, including drugs, physiotherapy, and group therapy. Moreover, children are not willing to do physiotherapy and group therapy because these treatments are uncomfortable and ineffective for them.

*“ … A treatment that shook her ears, she didn't want to do it and said it was uncomfortable … ” (Mother, p20)*

#### The advice to the health care service

In terms of treatment, many parents mentioned that the existing treatment is relatively simple and hoped to increase psychotherapy.

*“ … At present, it is mainly medication, and I think it still needs spiritual communication. At the psychological level, it is necessary to do some intervention and guidance … ” (Mother, p25)*

In addition, parents hoped that psychiatrists and nurses could communicate more effectively with their children in order to better understand their ideas. Many parents expressed concerns regarding how to get along with their children in the future, hoping that psychiatrists or nurses would give them guidance on parenting.

*“ … I think they need to communicate more with the kids. Psychiatrists and nurses may be more likely to communicate with my daughter than we are. After they know it, they can teach me how to communicate with her … ” (Mother, p23)*

*“ … For example, when you encounter such a situation, how do I communicate with her because she has to go to school and she is not willing to go to school?...” (Mother, p38)*

Some participants said that they did not have a professional mental health care institution in their place of residence, so it was difficult to register and hospitalize their child.

*“ … I tried to register from August 27, and I couldn't register in the hospital until September 18. Then, we have been waiting for a bed from September 18 to October 9th … ” (Mother, p32)*

## Discussion

We investigated the current Chinese mental health system from both the parents’ and medical staff’s perspective, via their experiences and perceptions when an adolescent with SRBs was admitted to the hospital. We found that China’s mental health resources are insufficient, resulting in low satisfaction with patients’ responses to treatment and high levels of stress for medical staff. In addition, a mature intervention system for SRBs is lacking.

Our study indicated a large number of barriers in the process of help-seeking for adolescents with SRBs. Most parents lack related knowledge and awareness of mental health. Previous research found that when parents know their children engaged in self-harm, their first reaction is to ignore [29]. Thus, until their children have serious SRBs, professional institutions were the last resort for parents. Moreover, insufficient resources for mental health services is another major problem. A cross-sectional study [30] showed that the average number of psychiatric beds, psychiatrists, and nurses was 3.15 / 100,000 population, 2.19 / 100,000 population, and 5.51 / 100,000 population, respectively, in China, lower than international average levels. Far worse, some areas lack mental health facilities altogether. Therefore, families need to see a doctor in more developed cities. The long-distance and the difficulty of registration in hospitals make it difficult for patients to receive timely treatment. In addition, patients with mental illness have commonly experienced social exclusion and stigmatization for many years [31]. This may also be one of the potential factors for patients to delay treatment.

Currently, there are no universally agreed-upon best practices for SRBs [32]. Although dialectical behavior therapy (DBT), cognitive-behavioral therapy (CBT), and mentalization-based therapy (MBT), mobile message intervention has a partial effect on the prevention of SRBs, their effectiveness in routine clinical settings still needs critical, additional research [32, 33]. In China, the treatment for SRBs is limited, with drug therapy the main treatment, supplemented by psychotherapy. However, drugs usually target mental illnesses that are comorbid with SRBs, and the effect of treatment is not significant; there may also be numerous side effects [34]. Moreover, the guidelines for the management of self-harm in young people and the latest systematic review [31, 35] exist that indicate that hospitalization is not the primary intervention for SRBs in adolescents and is not effective. Nevertheless, parents trust health professionals and have high expectations for their child’s cure. This expectation may create a large psychological gap for parents and affect return visits. In addition, our study found that the psychological treatments were all conducted separately, lacking a complete set of treatment models to ensure continuity. Notably, the psychotherapy in the wards is mainly implemented by nurses with few professional psychotherapists, and most of the nurses were educated through self-study. Hence, the level of psychotherapy is uneven and inconsistent.

Beyond that, the provision and coordination of good quality care in the mental health field represents a major challenge worldwide [36]. Our results showed that psychiatrists and nurses were understaffed and given demanding tasks, which resulted in less time spent communicating with patients and their families and less attention paid to mild inpatient care. In addition, parents are given little treatment information [37]. This is not conducive to the establishment of a good doctor-patient and nurse-patient relationship and has a certain impact on a patient’s re-visit and a parent’s understanding of the disease. On the other hand, the study indicates that negative perceptions towards teenagers exhibiting SRBs are common among medical staff. This is because psychiatric patients have a high risk of suicide and self-harm, high mood swings, and little desire for treatment. Hence, it is suggested that forming multidisciplinary mental health service teams (including psychiatrists, nurses, psychotherapists, psychological counselors, social workers, and rehabilitation specialists), strengthening the training of medical staff, and adding organizational support is needed to overcome these challenges.

### Limitations

There were some limitations to our study. First, the study was conducted in a general hospital, which may not fully reflect the situation of psychiatric hospitals. A second weakness is that retrospective assessment may reduce accuracy. A major limitation of this study was the failure not to include the adolescent patients’ perspective in the research, future researches could explore the adolescents’ experience.

## Conclusions

This study points out some problems in China’s mental health system and its treatment of SRBs. The public knows little about SRBs, and there is a shortage of mental health resources in China, resulting in many teenagers unable to see a doctor in time. We suggest increasing the input of mental health resources to expand and train mental health staff and establishing a complete set of treatment models for SRBs. In addition, it may also be necessary to try more interventions in the ward.

## Data Availability

The datasets used and/or analyzed during the current study are available from the corresponding author on reasonable request.

## Abbreviations

SRBs: Suicide-related behaviors
DBT: Dialectical behavior therapy
CBT: Cognitive-behavioral therapy
MBT: Mentalization-based therapy
